# The Contribution of Executive Functions to Academic Achievement in Gifted Children: A Cross-Sectional Study

**DOI:** 10.3390/jintelligence14030044

**Published:** 2026-03-09

**Authors:** Tindara Caprì, Giada Benedetta Catalano, Rosa Angela Fabio

**Affiliations:** 1 Department of Human Sciences, Link Campus University, Via del Casale di S. Pio V, 44, 00165 Rome, Italy; 2 Department of Biomedical and Dental Sciences and Morphofunctional Imaging, A.O.U. Policlinico Universitario “G. Martino” Via Consolare Valeria, 1, 98125 Messina, Italy; g.catalano@gmail.com (G.B.C.); rafabio@unime.it (R.A.F.)

**Keywords:** executive functions, metacognition, giftedness, academic achievement, planning, reading skills

## Abstract

Growing evidence indicates that executive functions, metacognition, and reading comprehension are crucial for academic success; however, their contribution to academic achievement in gifted children remains insufficiently understood. The main aim of this study was to compare planning processes and metacognitive abilities among gifted children with high academic achievement, gifted children with low academic achievement, and typically developing children with high academic achievement. A secondary aim was to examine reading comprehension in gifted children compared to typically developing peers. Seventy-three children (34 males, 39 females), aged between 8 and 11 years (M = 9.5, SD = 0.91), were divided into three groups: gifted children with high academic achievement, gifted children with low academic achievement, and typically developing children. Participants completed the Tower of London task, the MT Reading Comprehension Test, and the Me and My Mind metacognition questionnaire. Results showed that both groups of gifted children performed significantly better than typically developing peers in planning efficiency and reading comprehension. No significant differences emerged between high- and low-achieving gifted children in planning, reading comprehension, or metacognition. Overall, the findings suggest that planning abilities and reading comprehension represent cognitive strengths that distinguish gifted children from typically developing high achievers, whereas differences in academic achievement within the gifted population may be more closely related to metacognitive regulation and other non-cognitive factors rather than to planning or reading comprehension alone.

## 1. Introduction

The term intellectual giftedness refers to high intelligence quotient (Thompson & Oehlert, 2010). Children who exhibit exceptional intellectual abilities are identified through standardized intelligence tests, where they score two standard deviations above the mean IQ (above 130), with an estimated prevalence of 2.3% in the general population (Carman, 2013; Fabio, 2019; Fabio & Buzzai, 2019; McClain & Pfeiffer, 2012; Warne, 2015; Worrell et al., 2019). Gifted children typically demonstrate rapid development in various areas such as physical, cognitive, linguistic, and social areas (Bucaille et al., 2022). It is important to note that high intelligence is not the only distinguishing characteristic of giftedness but is also associated with superior performance in other cognitive abilities (Aubry et al., 2021; Bucaille et al., 2022). Research suggests that gifted children possess more complex, structured, and efficient cognitive abilities in information processing (Bucaille et al., 2022; Rocha et al., 2020) and typically show accelerated development in verbal skills and critical thinking, achieving higher academic results compared to typically developing children (Aubry et al., 2021; Fabio et al., 2022; Tibken et al., 2022).

Overall, intellectually gifted children tend to excel academically, especially in tasks that require a high level of executive control (Arffa, 2007; Liu et al., 2011a, 2011b). Gifted children are commonly defined as those who perform within the top 5% on standardized academic achievement tests (Kuznetsova et al., 2024), reflecting exceptional cognitive and scholastic abilities relative to their peers. However, high intellectual potential does not always translate into uniformly high academic performance. Not all gifted children fully express their academic potential; gifted children who achieve lower academic results than expected represent an exception to this trend (Tibken et al., 2022). A recent review has highlighted the existence of gifted children who demonstrate lower-than-expected academic achievement (Tourreix et al., 2023), suggesting that factors beyond general intelligence contribute to educational and academic outcomes. Therefore, it is possible to distinguish between gifted children with high academic achievement and those with low achievement (Barbier et al., 2019; Fabio, 2007).

Some studies have sought to explain the reasons underlying the differences in academic achievement despite a high intellectual quotient in these children (Hesam & Abedi, 2020; Leana-Taşcılar, 2024). The low achievement of gifted children can be attributed to various factors, including perfectionism, learned helplessness, socio-emotional issues (such as low motivation and self-efficacy), cultural and familial influences, as well as dual exceptionality—meaning the presence of disabilities or disorders that increase vulnerability to lower academic outcomes (Fong et al., 2023; McCoach & Siegle, 2003). Finch et al. (2014) have suggested that the low achievement may be linked to deficits in executive functions (EFs), but only few studies have closely examined EFs of these children.

Understanding the cognitive and self-regulatory processes that may account for this discrepancy is therefore of both theoretical and practical importance. The purpose of the present study was to offer a contribution to this research question.

### 1.1. EFs, Metacognition and Reading Comprehension in Academic Achievement

In educational context, academic achievement refers to the ability of students to handle and manage the demands of the study process and achieve good learning outcomes (Farooq et al., 2011). The most common approaches to measure academic achievement are the grade point average (GPA) and cumulative grade point average (CGPA) (York et al., 2015). The grade point average is calculated from grade points (or marks) that students achieve during their study. The cumulative grade point average is calculated as the average of all learned grade points divided by the possible number of points. These measurements have been considered the best single indicators of academic performance, providing both intelligibility and comparability (Prevatt et al., 2011).

Among the processes frequently associated with academic success are executive functions (EFs) and metacognition. EFs are typically conceptualized as a set of higher-order cognitive processes that support goal-directed behavior, including core components such as working memory, inhibitory control, and cognitive flexibility. Contemporary models emphasize both the unity and diversity of these components, underscoring their coordinated yet distinguishable contributions to complex learning tasks. Metacognition, broadly defined as knowledge about and regulation of one’s own cognitive processes, encompasses skills such as monitoring, evaluation, and planning.

Despite their conceptual proximity, the relationship between EFs and metacognition remains debated. Some theoretical perspectives (Hughes, 2019) propose that metacognition can be understood as a higher-order component within the broader framework of executive functioning. In contrast, other accounts argue that metacognition represents a partially independent construct, distinguishable from core executive processes in both structure and developmental trajectory. Moreover, certain strategies—such as planning—are frequently described as executive functions while also being classified as metacognitive strategies, further blurring conceptual boundaries.

Given these theoretical ambiguities, it is essential to frame the different measures under a clear conceptual model. It is well known that EFs comprise a series of complex cognitive processes enabling the control of thought and behavior (Monette et al., 2011; Vogelaar et al., 2017, 2019). However, in this study, planning is clearly considered as a metacognitive strategy.

Within this theoretical perspective, planning is described as the ideation of a mental representation of a given problem and the selection of an appropriate problem-solving strategy (Gligorovic & Buha, 2016), and it is suggested to be correlated with various other basic and higher-order EFs and believed that it can predict academic outcomes (McCormack & Atance, 2011; Zook et al., 2004). As regards gifted children, some studies suggested that planning abilities of gifted children were better developed than those of typically developing children (Zook et al., 2004). It was found that gifted children seem to outperform typical children in tasks involving executive control (Arffa, 2007; Liu et al., 2011a, 2011b).

When considering gifted children, it is also important to take into account that the development of EFs may follow a partially asynchronous and more heterogeneous trajectory. It is well established that EF development begins very early in life, with middle and late childhood representing a critical period due to the substantial neurodevelopmental changes in the prefrontal cortex during this stage (Bausela Herreras, 2024). However, a recent study found that, in the last stage of primary school, developmental changes are more evident in planning, whereas other EFs, such as inhibitory control, appeared comparatively stable, suggesting a differentiated pattern of EF development that may be further amplified in gifted children (Segundo-Marcos et al., 2022).

Closely related to EFs is metacognition, which represents a high-level cognitive process related to the control and regulation of our mental functioning, applied to learning and problem-solving (Straka et al., 2021). It mainly comprises three fundamental elements: metacognitive knowledge, cognitive monitoring, and regulation of problem-solving strategies. One of the functions of metacognition is to dictate how to perform one or more tasks and then ensure that they have been executed correctly. These executive processes include planning, evaluation, and regulation of problem-solving activities.

Several studies have suggested that gifted children have shown greater effectiveness in the use of metacognitive regulation strategies compared to their peers with average intellectual abilities, showing greater skill in defining, correcting, redefining, and solving problems (Swanson, 1990).

Closely related to EFs and academic achievement are reading comprehension abilities (Nouwens et al., 2021). According to the Simple View of Reading Model, reading comprehension depends on two fundamental components: decoding and linguistic comprehension (Gough & Tunmer, 1986). Reading comprehension refers to the process of interpreting words and connected speech and requires the activation of control processes and metacognitive knowledge.

Gokaydin et al. (2017) has highlighted that gifted children can show an early interest in reading, with notable abilities in comprehension compared to typically developing children. The advanced linguistic abilities of gifted children are considered a reflection of their superior mental capacities (Lesecq et al., 2024). Compared to typically developing children, gifted children have a more developed vocabulary, acquire reading skills at an early age, and construct longer, more meaningful sentences. However, research in this area still shows significant gaps. A recent literature review highlights the need for future studies to explain more in depth the superior abilities in reading comprehension observed in gifted children (Attoni et al., 2020).

Reading comprehension represents a particularly relevant construct when examining the relationship between EFs and academic achievement in gifted children. Unlike basic decoding skills, reading comprehension requires the coordinated engagement of multiple higher-order cognitive processes, including working memory (to maintain and integrate textual information), inhibitory control (to suppress irrelevant interpretations), cognitive flexibility (to revise meaning in light of new information), and planning and monitoring processes typically associated with metacognition. As such, reading comprehension can be conceptualized as an ecologically valid and academically meaningful outcome that reflects the functional integration of executive and metacognitive processes. This is especially pertinent in gifted populations, for whom decoding skills are often well developed, making comprehension a more sensitive indicator of higher-order cognitive regulation. Moreover, variability in reading comprehension performance among gifted children may illuminate how differences in executive and metacognitive functioning contribute to discrepancies between intellectual potential and observable academic achievement.

### 1.2. The Present Study and Research Questions

The literature strongly suggested that EFs predict academic outcomes, with some research showing EFs is a better predictor of reading comprehension than IQ alone (Diamond, 2013). Although gifted children typically exhibit strong executive functioning skills, they may nonetheless display either high or low levels of academic achievement. Therefore, it was unclear how EFs can predict academic achievement in gifted children. Thus, some questions are still open in the literature: whether there are differences in EFs of gifted children with high or low academic achievement and whether the potential differences in EFs are positively related to high or low academic achievement.

The present study aimed to offer a contribution to the above questions. The main aim of this study was to compare planning processes and metacognitive abilities among gifted children with high and low academic achievement and typically developing (TD) children with high academic achievement. Given that reading comprehension is related to EFs and can affect the level of academic achievement, the second aim of this study was to examine reading comprehension in gifted children compared to typically developing children.

It was hypothesized that significant differences existed among the groups under consideration. First, to confirm the literature date, it was hypothesized that gifted children with high achievement showed a better performance in planning abilities, reading comprehension and metacognition compared to typically developing children with high academic achievement. Second, it was hypothesized that gifted children with high achievement performed better than gifted children with low achievement. Third, it was hypothesized that gifted children with low achievement demonstrated a higher performance in planning abilities, reading comprehension and metacognition compared to typically developing children with high academic achievement.

## 2. Materials and Methods

### 2.1. Participants

The initial pool consisted of 1123 children attending mainstream public primary schools located in Southern Italy. Schools were contacted through institutional collaboration with school principals and teachers, and participation was voluntary. The sample therefore represents a convenience sample of students from general education classrooms rather than a population-based sample, and no snowball recruitment procedures were used. All children were screened to identify potential intellectual giftedness using the Colored Progressive Matrices. Demographic information (age, sex, and socioeconomic status) was collected through school records and parent reports.

The inclusion criteria were: aged from 6 to 11 years, absence of psychological, neurological disorders and/or Learning Disorders, and high/low academic achievement. The exclusion criteria were current psychological or pharmacological treatment and Intellectual Disability. During the initial screening, a psychologist conducted a clinical interview to verify the absence of developmental or clinical conditions that could affect participation. Based on the screening phase, 58 participants obtained scores above the 95th percentile on the Colored Progressive Matrices (CPM; Raven, 2000). The 95th percentile on the CPM was adopted as the cutoff criterion to classify giftedness in order to ensure a stringent and widely recognized threshold for identifying giftedness. In psychometric research, performance at or above the 95th percentile is commonly used to operationalize giftedness, as it corresponds to the upper extreme of the normative distribution and reflects significantly above-average cognitive ability. Using the 95th percentile as a cutoff allowed us to identify children whose reasoning abilities clearly exceeded age-based norms while minimizing the influence of linguistic, educational, or socio-cultural factors. This criterion ensured methodological rigor and consistency with previous research employing non-verbal measures to operationalize giftedness in school-aged populations (Kuznetsova et al., 2024).

The CPM were selected as the screening instrument because they provide a well-validated, culture-fair measure of non-verbal fluid reasoning, which represents a core component of general intellectual ability. Although the definition of giftedness presented in the introduction refers to high intellectual functioning more broadly, the use of CPM allowed us to minimize the influence of language, socio-cultural background, and prior schooling on performance. This was particularly relevant given the young age of the participants and the focus on executive and metacognitive processes, which could be confounded by verbally loaded IQ measures. Furthermore, CPM is widely used in research settings as a reliable proxy for general cognitive ability and has demonstrated strong correlations with full-scale IQ indices. Therefore, it provided an appropriate and methodologically consistent criterion for identifying children with giftedness within the constraints of the study design.

These 58 participants with giftedness were divided into two groups based on their academic achievement: 39 gifted children with high academic achievement (19 males and 20 females) and 19 gifted children with low achievement (9 males and 10 females). In addition, four children presented intermediate GPA values (6.00–8.99) and were excluded from the study as they did not meet the criteria for either high or low academic achievement.

Academic achievement was measured with the Grade Point Average (GPA), which is a numerical representation of a student’s academic performance, calculated by averaging the grades earned across all courses. Typically, primary schools in Italy use a ten-point numerical grading scale, where 6 is the minimum passing grade and 10 is the highest. To classify these children into “high” and “low” academic achievement, we used the following criteria: high academic achievement was defined as a GPA score ranging from 9 to 10, whereas low academic achievement was defined as a GPA score below 6.

From the remaining screened school sample (i.e., children who did not meet the giftedness criterion), 15 typically developing children with high academic achievement (6 males and 9 females) were recruited and included in the study as a comparison group. The final sample consisted of 73 participants (34 males and 39 females), aged between 8 and 11 years (M = 9.5; SD = 0.91), divided into three groups: gifted group with high academic achievement (GHA), gifted group with low academic achievement (GLA), and typically developing group with high academic achievement (TD). Although the screening phase targeted children aged 6–11 years, the final analyzed sample comprised children aged 8–11 years. All participants were Italian and were native Italian speakers.

**Figure 1:**
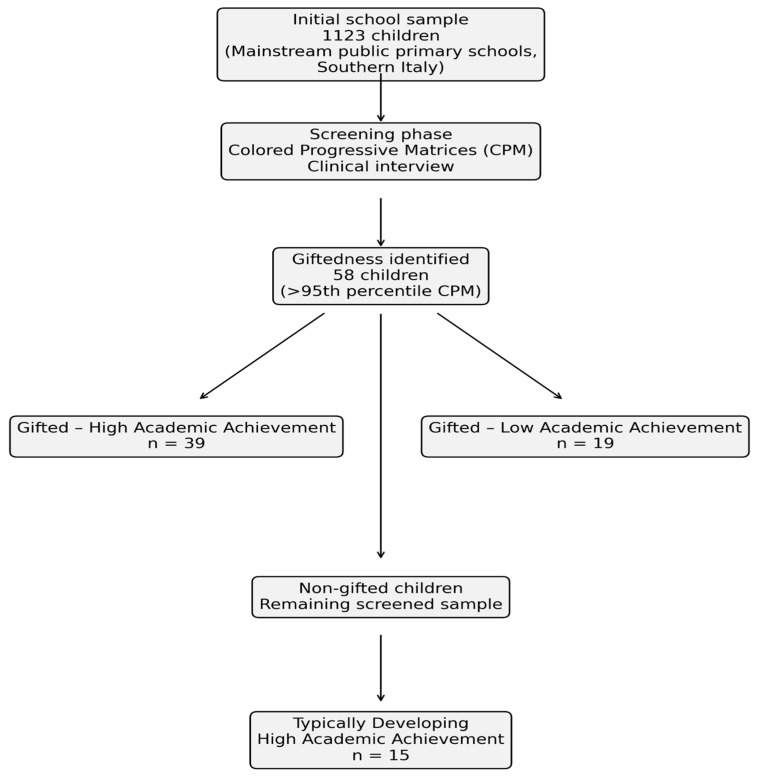
Flow diagram of the recruitment, screening, and selection procedure. From an initial school-based convenience sample of 1123 children attending mainstream public primary schools in Southern Italy, 58 children met the criterion for intellectual giftedness (>95th percentile on the Colored Progressive Matrices) and were divided into gifted children with high academic achievement (*n* = 39) and gifted children with low academic achievement (*n* = 19). From the remaining non-gifted students, a comparison group of typically developing children with high academic achievement (*n* = 15) was recruited, resulting in a final sample of 73 participants.

The participant recruitment and selection process is summarized in Figure 1. Table 1 shows demographic characteristics of participants for each group.

### 2.2. Measurements

The Colored Progressive Matrices (CPM; Raven, 2000) were used to measure general intellectual functioning in the screening phase to recruit gifted children. The Tower of London (TOL; Boccia et al., 2017) was used to evaluate planning abilities. The MT Comprehension test (Cornoldi & Colpo,2006) was used to evaluate decoding skills and reading comprehension. The “Me and My Mind” metacognition questionnaire (Friso et al., 2006) was used to measure metacognition. To measure academic achievement, the Grade Point Average was used.

Colored Progressive Matrices (CPM). This test comprises a booklet containing 36 colored matrices, divided into three sets of 12 items each (Matrices A, Ab, and B), characterized by increasing levels of difficulty. Matrice A assesses the ability to analyze identities, similarities, and differences; Matrice Ab focuses on spatial orientation (symmetry and position), while Matrice B tests logical and spatial principles (Raven, 2000). Each item consists of a colored pattern presented on an A4 sheet: the upper part of the sheet displays a matrix with a missing portion, while the lower part provides six options to choose from to complete the matrix. The 36 matrices were presented in a specific sequence: matrice A, and then AB, and finally B. The final score consists of the total number of correct responses that correspond to specific percentile scores according to normative data. The final score indicates general intellectual functioning. The psychometric properties of the test are very good, with a test–retest reliability generally around 0.90 (Raven, 2000) and a Cronbach’s alpha coefficient of 0.91 (Belacchi et al., 2008). In the present sample, the internal consistency of the CPM was high (Cronbach’s α = 0.85).

Tower of London Test (TOL). This test was employed to assess EFs, particularly planning and problem-solving. It involves presenting the participant with a series of configurations of colored discs arranged on three pegs of varying heights. The goal is to move the discs from an initial arrangement to a final one following specific rules, using the fewest possible number of moves. The test includes different levels of difficulty, with a gradual increase in the complexity of the configurations. Both the quality of planning and the time taken to complete each configuration are evaluated. The TOL is widely used in assessing cognitive functions in various clinical and research settings (Boccia et al., 2017). The total number of perfect solutions was recorded. For each problem set, the average number of moves to complete the set, initial planning time, total execution time and rule violations were calculated.

MT Comprehension Test. This test was used to assess reading comprehension in primary and middle schools (Cornoldi & Colpo, 2006). The test requires the student to read a passage and then answer a series of multiple-choice questions (10 for primary and 10 for middle schools). Responses are used to establish the student’s comprehension level. This test allows students to be assigned to one of five performance categories: standard achieved, good level, sufficient, insufficient, or seriously insufficient.

Me and my mind metacognition questionnaire. This test is composed of 15 items called “metacognitive dilemmas,” each accompanied by a graphic representation and four response options. For example, an instance of this tool might ask the child: “If you have to remember a story, is it easier to...” The response options are (a) remember it by heart, (b) remember exactly what each character says, (c) remember what happens in your own words, and (d) remember the exact order of events. The average time for completion is about 20 min. The final score consists of the total number of correct responses obtained. The questionnaire has demonstrated good internal consistency in previous research (α = 0.67; Friso et al., 2006) and has been standardized on a sample of Italian elementary school students in grades three through five. In the present sample, internal consistency was acceptable (Cronbach’s α = 0.71).

Grade Point Average (GPA). A GPA is a numerical representation of a student’s academic performance, calculated by averaging the grades earned across all courses. Typically, primary schools in Italy use a ten-point numerical grading scale, where 6 is the minimum passing grade and 10 is the highest grade. The GPA is a numerical average that reflects a student’s overall academic achievement. In addition to its use for group classification, continuous GPA values were also used as a quantitative indicator of academic achievement in correlation and regression analyses.

### 2.3. Procedure

Participants were tested in an individual setting within the school classrooms of the institutions involved. The administration of tests occurred randomly and required a total time of 90 min. Informed consent was obtained from participants’ parents.

### 2.4. Study Design

The present study adopted a cross-sectional design, examining academic achievement, metacognition, planning, and reading comprehension at a single time point in gifted children and TD children. Given the developmental nature of these constructs in developmental age, a cross-sectional study allows for examining associations between many variables at a single point in time, offering a snapshot of gifted children with low-high academic achievement without the high costs or follow-up logistics associated with longitudinal studies.

### 2.5. Statistical Analysis

Data were analyzed using SPSS version 24.0 for Windows. Descriptive statistics were computed for all study variables. The alpha level was set at 0.05. Assumptions were examined via descriptive indices and visual inspection (histograms, Q–Q plots), and no substantial violations were observed.

Group differences across planning (Tower of London indices), reading comprehension, and metacognition were tested using one-way ANOVAs with Group (GHA, GLA, TD) as the between-subject factor. Homogeneity of variance was assessed via Levene’s test; when met, Tukey’s HSD post hoc comparisons were performed. Effect sizes are reported as partial eta squared (ηp^2^).

Pearson correlations were conducted using continuous scores. GPA was used categorically only for group classification within the gifted sample, whereas continuous GPA values were used in correlational analyses.

## 3. Results

To examine differences among the three groups (GHA, GLA, TD), a series of one-way analyses of variance (ANOVAs) were conducted with Group as the between-subject factor. When significant main effects emerged, Tukey’s HSD post hoc comparisons were performed. Effect sizes are reported as partial eta squared (ηp^2^). Descriptive statistics for all variables are presented in Table 2. The overall ANOVA results are summarized in Table 3.

### 3.1. Group Differences

#### 3.1.1. Tower of London (Planning Measures)

A significant group effect was found for TOL initial planning time, *F*(2,70) = 7.21, *p* = .006, ηp^2^ = 0.11. Post hoc comparisons indicated that the TD group (M = 437.19, SD = 178.79) showed significantly longer planning times compared to both the GHA group (M = 301.80, SD = 155.40; *p* = .009) and the GLA group (M = 277.44, SD = 73.55; *p* = .02). No significant difference emerged between the two gifted groups (*p* > .40). For TOL execution time, the ANOVA revealed a non-significant effect, F(2,70) = 2.45, *p* = .090, ηp^2^ = 0.07. Although descriptively the TD group showed longer execution times, post hoc comparisons were not statistically significant. For TOL number of perfect solutions, a significant group effect emerged, F(2,70) = 3.60, *p* = .032, ηp^2^ = 0.09. Post hoc analyses indicated that both gifted groups (GHA: M = 28.95; GLA: M = 28.73) performed significantly better than the TD group (M = 26.93; *p* < .05), whereas no significant difference was found between GHA and GLA (*p* > .80). No significant group differences were observed for TOL total number of moves (F(2,70) = 0.85, *p* = .430) and rule violations (F(2,70) = 0.12, *p* = .880).

#### 3.1.2. Reading Comprehension

A significant group effect was found for MT Reading Comprehension, F(2,70) = 4.20, *p* = .019, ηp^2^ = 0.11. Post hoc comparisons indicated that both the GHA (M = 1.43, SD = 1.50) and GLA (M = 1.70, SD = 1.16) groups scored significantly higher than the TD group (M = 0.93, SD = 0.73; *p* < .05). No statistically significant difference emerged between the two gifted groups (*p* > .40).

#### 3.1.3. Metacognition

The ANOVA for metacognition did not reveal a significant group effect (F(2,70) = 1.05, *p* = .350, ηp^2^ = 0.03), indicating comparable scores across the three groups.

### 3.2. Direct Comparison Between the Two Gifted Groups

Direct post hoc comparisons between GHA and GLA across all outcome measures are presented in Table 4. No statistically significant differences were observed between the two gifted groups in planning measures, reading comprehension, or metacognition (all adjusted *p* > .40). Effect sizes were small (Cohen’s d ranging approximately from 0.05 to 0.45), indicating highly comparable cognitive profiles between high- and low-achieving gifted children within the present sample.

### 3.3. Correlations

Pearson correlation analyses were conducted using continuous scores (Table 5). Planning indicators were moderately intercorrelated, particularly initial planning time and total execution time (r = 0.68, *p* < .01), suggesting that these measures capture related aspects of planning efficiency. Reading comprehension showed a small but significant positive association with planning efficiency (r = 0.29, *p* < .05). With respect to academic achievement, GPA was significantly correlated only with metacognition (r = 0.33, *p* < .05), whereas correlations with planning and reading comprehension were small and non-significant. Overall, the magnitude of the associations was modest, supporting the view that academic achievement is influenced by multiple interacting cognitive and non-cognitive factors.

### 3.4. Predictive Contribution to Academic Achievement

To directly examine the contribution of planning, reading comprehension, and metacognition to academic achievement, a multiple linear regression analysis was conducted using continuous GPA as the dependent variable. Among the Tower of London indices, initial planning time was selected as the primary indicator of planning efficiency because it most directly reflects anticipatory strategic processing prior to task execution and showed the strongest group effect in the ANOVA results. This choice also allowed for a more parsimonious model, avoiding multicollinearity among highly intercorrelated Tower of London indices. Metacognition, MT reading comprehension, and TOL initial planning time were entered simultaneously as predictors. As shown in Table 6, the overall model accounted for approximately 11–13% of the variance in GPA (R^2^ = 0.11–0.13, *p* < .05). Metacognition emerged as the only significant independent predictor of academic achievement (positive association), whereas reading comprehension and planning did not account for significant additional variance. These findings indicate that, within the present sample, metacognitive abilities show the most consistent unique association with academic achievement when controlling planning efficiency and reading comprehension. Regression coefficients are presented in Table 7. Metacognition emerged as the only significant independent predictor of GPA (β ≈ 0.31, *p* < .05), whereas MT comprehension and TOL initial planning time did not show significant unique contributions. These findings indicate that metacognitive abilities are the most consistent predictor of academic achievement within the present model.

## 4. Discussion

The present study aimed to examine planning abilities, reading comprehension, and metacognition in gifted children with high and low academic achievement, compared to typically developing (TD) children with high academic achievement. Overall, the findings partially supported the study hypotheses and provided a more nuanced picture of the cognitive factors associated with academic achievement within gifted populations.

With respect to the first hypothesis, results showed that gifted children with high academic achievement outperformed typically developing peers in planning efficiency (as reflected by initial planning time and number of perfect solutions on the Tower of London) and in reading comprehension. These findings are consistent with previous literature documenting superior executive functioning and advanced language-related skills in gifted children (Aubry et al., 2021; Bucaille et al., 2023; Rocha et al., 2020). In contrast, no group differences emerged in metacognition, suggesting that metacognitive skills, as assessed in the present study, did not distinguish gifted and typically developing high-achieving children.

Contrary to the second hypothesis, no statistically significant differences were detected between high- and low-achieving gifted children in planning abilities, reading comprehension, or metacognition. This finding diverges from studies suggesting that executive or self-regulatory weaknesses may underline academic underachievement in gifted students (Aubry et al., 2021; Bucaille et al., 2022). However, the absence of significant differences should not be interpreted as evidence of equivalence between gifted subgroups. Given the limited statistical power of the gifted-group comparisons, smaller effects cannot be excluded and should be examined in future studies with larger samples.

The third hypothesis was partially supported. Gifted children with low academic achievement demonstrated significantly better planning efficiency and reading comprehension than typically developing peers but did not differ from high-achieving gifted children on any of the assessed cognitive measures. This pattern suggests that planning and reading comprehension represent cognitive strengths associated with giftedness per se, rather than factors that clearly differentiate levels of academic achievement within the gifted population.

Taken together, these findings indicate that gifted children, regardless of academic achievement level, tend to exhibit stronger planning abilities and reading comprehension than typically developing peers with high academic achievement. This pattern aligns with previous research highlighting more efficient executive control and advanced linguistic processing in gifted populations (Aubry et al., 2021; Bucaille et al., 2023; Rocha et al., 2020). Planning, as a component of executive functioning, supports goal setting, task organization, and strategic problem solving, and may facilitate more efficient engagement with complex cognitive tasks. Similarly, superior reading comprehension in gifted children may reflect advanced vocabulary, inferential reasoning, and the ability to integrate information across text segments.

Regarding metacognition, no group differences were observed among gifted and typically developing children. This result suggests that metacognitive skills, as measured by the self-report questionnaire used in this study, may not differentiate these groups at this developmental stage. Nonetheless, correlational analyses revealed a positive association between metacognition and academic achievement, indicating that metacognitive regulation may still play an important role in supporting school performance across groups.

Importantly, the multiple linear regression analysis provided a more direct examination of the relative contribution of the assessed variables to academic achievement. When metacognition, reading comprehension, and planning efficiency (indexed by Tower of London initial planning time) were entered simultaneously as predictors of continuous GPA, metacognition emerged as the only significant independent predictor. Planning and reading comprehension did not account for additional unique variance. Although the overall variance explained was modest, this finding suggests that metacognitive abilities may represent a more consistent contributor to academic achievement than the executive and reading measures assessed here, particularly when multiple cognitive factors are considered simultaneously.

The relatively small magnitude of the observed correlations and the modest explanatory power of the regression model likely reflect the multifactorial nature of academic achievement. Cognitive abilities interact with motivational, emotional, and contextual factors, which were not directly assessed in the present study. Moreover, the relatively small sample size may have limited the detection of subtle associations, particularly within gifted subgroups. Future research with larger samples should explore group-specific relationships among executive functions, metacognition, and academic achievement.

From an applied perspective, the findings suggest that interventions aimed at supporting gifted students should not focus exclusively on cognitive skills such as planning or reading comprehension, particularly for students with lower academic achievement. Instead, fostering metacognitive regulation, motivation, and self-regulatory learning strategies may be especially relevant for helping gifted children translate cognitive potential into consistent academic success.

## 5. Conclusions

The present study examined whether planning abilities, metacognition, and reading comprehension differentiate gifted children with high and low academic achievement from typically developing high-achieving peers, and how these variables relate to academic performance. The findings highlight both the cognitive strengths associated with giftedness and the complexity of factors influencing academic trajectories within this population.

Gifted children, regardless of academic achievement level, demonstrated more efficient planning abilities and higher reading comprehension than typically developing peers with high academic achievement. In contrast, no differences emerged in metacognition across groups. Moreover, no statistically significant differences were detected between high- and low-achieving gifted children in planning, reading comprehension, or metacognition; however, given the limited statistical power, smaller effects cannot be excluded.

Importantly, planning abilities distinguished gifted children from typically developing peers but did not differentiate levels of academic achievement within the gifted group. Reading comprehension showed a similar pattern, suggesting that these cognitive domains may reflect core characteristics of giftedness rather than mechanisms underlying underachievement. In contrast, metacognition was positively associated with academic achievement and emerged as the only significant independent predictor of GPA in the regression model, underscoring its potential role as a key regulatory factor supporting school success.

Overall, these findings suggest that academic underachievement in gifted children may not be adequately explained by differences in planning abilities or reading comprehension alone. Instead, non-cognitive and self-regulatory factors—such as metacognitive monitoring, motivation, engagement, and socio-emotional variables—may play a more decisive role in shaping academic outcomes. Future longitudinal studies with larger samples and more sensitive measures are needed to clarify how these factors interact over time to influence academic trajectories in gifted populations.

## Figures and Tables

**Table 1:** Demographic characteristics of three groups.

Groups	Measures	Values
GHA	*n*, male/female	19/20
	Age, *M*/(*SD*)	9.5 (0.91)
	Socioeconomic status, *n*	
	Lower Class	0
	Middle Class	37
	Upper Class	3
GLA	*n*, male/female	9/10
	Age, *M*/(*SD*)	9.5 (0.91)
	Socioeconomic status, *n*	
	Lower Class	0
	Middle Class	17
	Upper Class	2
TD	*n*, male/female	6/9
	Age, *M*/(*SD*)	9.5 (0.91)
	Socioeconomic status	
	Lowe Class	0
	Middle Class	6
	Upper Class	3

**Table 2:** Mean (M) and standard deviation (SD) of all analyzed parameters for the three groups.

Parameters	Groups					
	GHA		GLA		TD	
	M	SD	M	SD	M	SD
TOL number of moves	218.90	162.91	245.68	152.83	260.26	170.08
TOL initial planning time	301.80	155.40	277.44	73.55	437.19	178.79
TOL execution time	531.19	228.79	518.55	182.69	642.96	320.89
TOL rule violations	3.46	3.21	3.36	2.20	3.21	1.76
TOL number of perfect solutions	28.95	3.87	28.73	2.57	26.93	3.02
MT Comprehension Test	1.43	1.50	1.70	1.16	0.93	0.73
Metacognition	9.20	2.42	8.20	1.87	8.79	2.01

**Table 3:** One-way ANOVA results for group differences.

Variable	F (2,70)	*p*	ηp^2^
TOL Initial Planning Time	7.21	.006	0.11
TOL Execution Time	2.45	.090	0.07
TOL Perfect Solutions	3.60	.032	0.09
TOL Number of Moves	0.85	.430	0.02
TOL Rule Violations	0.12	.880	0.003
MT Comprehension	4.20	.019	0.11
Metacognition	1.05	.350	0.03

**Table 4:** Direct comparison between GHA and GLA groups.

Variable	Mean Difference (GHA–GLA)	Cohen’s d	Adjusted *p*
TOL Initial Planning Time	24.36	0.20	.650
TOL Execution Time	12.64	0.06	.780
TOL Perfect Solutions	0.22	0.07	.850
TOL Number of Moves	−26.78	0.17	.600
MT Comprehension	−0.27	0.22	.400
Metacognition	1.00	0.45	.120

Note: Adjusted *p* values refer to Tukey’s HSD post hoc comparisons.

**Table 5:** Correlations between considered parameters.

Variable	1	2	3	4	5	6	7
1. TOL Initial Planning Time	—						
2. TOL Execution Time	0.68 **	—					
3. TOL Rule Violations	−0.25	0.10	—				
4. TOL Perfect Solutions	0.05	−0.44 **	−0.37 **	—			
5. Reading Comprehension	−0.13	−0.19	−0.10	0.29 *	—		
6. Metacognition	0.01	−0.08	−0.14	0.19	0.05	—	
7. GPA	−0.06	0.11	−0.05	−0.02	0.02	0.33 *	—

* *p* < 0.05; ** *p* < 0.001.

**Table 6:** Hierarchical regression predicting GPA (continuous).

Step	Predictors Entered	R^2^	ΔR^2^	F Change (df)	*p*
1	Metacognition	0.11	0.11	F(1,71) = 4.14	.046
2	MT comprehension	0.11	0.003	F(1,70) = 1.12	.29
3	TOL initial planning time	0.11	0.004	F(1,69) = 1.11	.30

Final model: R^2^ = 0.11, Adjusted R^2^ = 0.07, F(3,69) = 2.83, *p* = .045.

**Table 7:** Regression coefficients predicting GPA (continuous).

Predictor	B	SE B	β	t	*p*
Metacognition	0.18	0.09	0.31	2.04	.045
MT comprehension	0.05	0.07	0.08	0.71	.48
TOL initial planning time	−0.0002	0.0003	−0.09	−0.82	.41

## Data Availability

The datasets that support the findings of this research are not publicly available due to the sensitive and identifying nature of the data, in line with the consent provided by participants. Data can be made available upon request by contacting the corresponding author T.C. (t.capri@unilink.it).
